# Effects of individualized administration of folic acid on prothrombotic state and vascular endothelial function with H-type hypertension: A double-blinded, randomized clinical cohort study

**DOI:** 10.1097/MD.0000000000028628

**Published:** 2022-01-21

**Authors:** Song Zhang, Tianxun Wang, Huaiqi Wang, Jianping Tang, Ailin Hou, Xiaoling Yan, Baozhong Yu, Shuangming Ran, Min Luo, Ying Tang, Ruohan Yang, Dongsheng Song, Hanjun He

**Affiliations:** The First People's Hospital of Guangyuan, Sichuan, China.

**Keywords:** folic acid, H-type hypertension, methylene tetrahydrofolate reductase C677T, personalized medicine, prothrombotic state

## Abstract

**Background:**

Hypertension and hyperhomocysteinemia (HHcy) have long been associated with adverse cardiovascular and cerebrovascular health outcomes. This study evaluated the effect of individualized administration of folic acid (FA) on homocysteine (Hcy) levels, prothrombotic state, and blood pressure (BP) in patients with H-type hypertension (combination of HHcy and hypertension).

**Methods:**

In this double-blinded, randomized clinical cohort study, 126 patients with H-type hypertension who were treated at our hospital were randomly divided into treatment and control groups (n = 55 each). The control group was treated with oral levamlodipine besylate tablets 2.5 mg and placebo, once a day (in the morning). The treatment group was first treated with oral levamlodipine besylate 2.5 mg and FA tablets 0.8 mg, once a day (in the morning), for 12 weeks. Then, in a second 12-week phase, the FA dose was adjusted using the methylene tetrahydrofolate reductase C677 polymorphism genotype. The levels of Hcy and coagulation factors, prothrombotic state parameters, BP, and adverse drug reactions were compared between the 2 groups.

**Results:**

Pretreatment general patient characteristics, including Hcy levels, were similar between the 2 groups (*P* > .05). BP and prothrombotic status did not differ before and after the first phase of treatment (*P* > .05). However, Hcy and endothelin-1 (ET-1) levels decreased, while nitric oxide levels increased significantly in the intervention group (*P* < .05). In the second phase, after 3 months’ treatment with an FA dose adjusted according to methylene tetrahydrofolate reductase C677T genotype, the Hcy and ET-1/NO levels were significantly decreased in the intervention group and were lower than those after the first treatment phase and lower than in the control group (*P* < .01). BP, D-dimer levels, and fibrinogen scores were significantly lower after the second treatment phase (*P* < .01). There was no significant difference in the incidence of adverse drug reactions between the 2 groups (*P* > .05).

**Conclusions:**

Individualized administration of FA tablets can effectively reduce BP, and Hcy and coagulation factor levels, and significantly improve prothrombotic status in patients with H-type hypertension.

## 1 Introduction

Hypertension is the leading cause of death worldwide, and its incidence is increasing.^[1]^ Hypertension and hyperhomocysteinemia (HHcy) have long been associated with decreased cardiovascular and cerebrovascular health outcomes. The combination of HHcy and hypertension, referred to as H-type hypertension, has been identified in 60% of people with hypertension, based on blood tests.

HHcy is associated with the methylene tetrahydrofolate reductase (*MTHFR*) C677T polymorphism, folic acid (FA), and an increased rate of atherosclerotic plaques.^[2,3]^ Many studies have indicated that HHcy is associated with a number of vascular and hematological changes, which create a proatherogenic and prothrombotic milieu.^[4,5]^ Recently, HHcy has been established as an independent risk factor for vascular occlusive diseases, such as arterial and venous thrombosis.^[4,5]^ HHcy is associated with a poor prognosis in terms of clinical outcomes, increased atherosclerotic plaque, and prothrombotic state.^[6]^ In patients with poorly controlled H-type hypertension, there is an increased risk of atherosclerotic plaque, decreased nitric oxide (NO) levels, and delayed recovery from a prothrombotic state.^[7]^ Thus, there is considerable evidence that homocysteine (Hcy) levels are an important prognostic factor for hypertension in individuals with a prothrombotic state.^[8]^

*MTHFR* is located on chromosome 1p36.3. Residue 677 can variably be cytosine (C) or thymidine (T). The *MTHFR* C677T genotype is categorized as homozygous CC, heterozygous CT, and homozygous TT. The TT genotype is associated with HHcy.^[9]^ The T allele has a high frequency in Chinese people.^[10–12]^

There is a paucity of evidence on FA levels in people with a prothrombotic state who have hypertension, although the association between HHcy and the prothrombotic state has been repeatedly demonstrated in evidence-based medicine. Calcium channel blockers (CCBs) are among the mainstays of treatment for hypertension. In addition to its antihypertensive effects, the CCB levamlodipine besylate has demonstrated cardiovascular protective effects. In addition to controlling blood pressure (BP), it is the best option for preventing or repairing such damage to atherosclerotic plaques by increasing NO levels.^[13]^ Therefore, it is reasonable to test whether treatment with FA and levamlodipine besylate can decrease Hcy levels to improve clinical endpoints.

This prospective, double-blind, randomized, controlled study investigated whether individualized administration of FA along with levamlodipine besylate can improve the prothrombotic state and vascular endothelial function in H-type hypertension patients.

## 2 Materials and methods

### 2.1 Study participants

In our double-blinded randomized controlled trial, we studied people with H-type hypertension and a prothrombotic state to determine the effectiveness of FA with levamlodipine besylate. A total of 126 patients with H-type hypertension were recruited from the First People's Hospital of Guangyuan in Sichuan Province, China from October 1, 2019 to October 31, 2020. All participants were recruited from the Department of Cardiology. The study was approved by the institutional review board of The First People's Hospital of Guangyuan and the patients provided written informed consent for participation in this two-phase study. Patients were recruited in the study if they had primary hypertension (systolic BP ≥ 140 mm Hg, diastolic BP ≥ 90 mm Hg), were aged over 18 years, were treated with levamlodipine besylate, had the capacity for providing informed consent, and reported having HHcy(>15 mmol/L). Participants were excluded if they were pregnant, had severe anxiety treated with antianxiety drugs, had hypertensive crisis, had a history of stroke, had other complications, such as diabetes, tumor, or myocardial infarction, or if agents that affect vitamin metabolism, such as methotrexate, niacin, and phenytoin, or FA, vitamin B12, antiepilepsy drugs, antituberculosis drugs, ethanol, or oral contraceptives had been taken in the past 2 months.

Patients’ baseline characteristics and BP measurements were recorded after they provided consent for participation.

### 2.2 Randomization

All patients were randomly allocated to the treatment and control groups by computer-based simple random sampling in a 1:1 ratio, without stratification, using EXCEL software (Microsoft Inc., Redmond, WA). In accordance with the random number table, the patients were randomly and equally allocated to groups receiving levamlodipine besylate and placebo (control group) or FA and levamlodipine besylate (treatment group). Randomization was performed after patients agreed to the baseline measurements after a structured discussion with the clinic doctor and the pharmacist at the baseline visit. Randomization was performed by an independent researcher with no involvement in the inclusion and exclusion process, treatment, or outcome assessments. The nurse, patients, and study pharmacist were blinded to group allocation. Group allocation was only accessible to the research pharmacist. All participants were advised of the potential side effects of FA and were advised to use individualized medication doses based on the *MTHFR* C677T test.

### 2.3 Groups

Both groups received levamlodipine besylate, while the treatment group received FA and the control group were given placebo. Both groups were blinded to group allocation. All participants were asked to maintain a diary of medication after their recruitment. The treatment group received 0.8 mg FA and 2.5 mg levamlodipine once per day (in the morning), whereas the control group received 2.5 mg levamlodipine and placebo. After the first phase of treatment (12 weeks), the dose of FA in the treatment group was adjusted by the *MTHFR* C677T genotype and treatment continued for another 12 weeks.

### 2.4 Outcome measures

Serum Hcy levels in patients with H-type hypertension were detected by enzymatic cycling assay. Enzyme-linked immunosorbent assay were used to detect ET-1 and NO levels. The prothrombotic state, fibrinogen and D-dimer levels were detected using a fully automated immunoassay. Activated partial prothrombin time and prothrombin time were measured using a fully automated blood coagulation analyzer. The *MTHFR* C677T polymorphism was analyzed using polymerase chain reaction-restriction fragment length polymorphism. The relationship between serum Hcy levels, ET-1, NO, and the index of vascular endothelial cell injury were analyzed. The enzymatic cycling assays, enzyme-linked immunosorbent assays, and polymerase chain reaction tests were performed at the clinical laboratory. These methods are simple, accurate, and reproducible, and provide a reference standard for clinical diagnosis.

### 2.5 Data collection

The patients were seen 3 times by the research assistant: at baseline, 12 weeks after treatment, and 12 weeks after adjusting the dose of FA according to the *MTHFR* C677T genotype. BP, prothrombotic state parameter measurements, and levels of Hcy, ET-1, and NO were recorded at each time point after treatment.

### 2.6 Data analysis

Data are displayed as the mean and standard deviation. Numbers and percentages are used to describe categorical data. To test if there were any effects from random differences at baseline after treatment with FA. The dependent variable was FA, and the covariants were Hcy, NO, ET-1, and prothrombotic state. The adjusted FA was performed after gene testing. For nominal data, independent Student *t* tests were used to compare the groups. A *P*-value < .05 was considered statistically significant. Statistical data were analyzed using SPSS version 19 software (IBM Inc., Armonk, NY).

## 3 Results

### 3.1 Flow of patients

A total of 126 patients were initially recruited and randomly allocated to the treatment and control groups. Only 110 patients completed the trial, as 16 patients were lost to follow-up (8 each in the treatment and control group). The clinical material flow diagram of the patients through the trial is displayed in the CONSORT 2010 Flow Diagram. Patient characteristics and baseline records are shown in Table 1. Clinical data were obtained for the majority of the baseline assessments (Table 1). Patient characteristics and baseline clinical details values did not differ significantly between groups (*P* > .05).

**Table 1:** Participants’ characteristics and baseline clinical details.

Randomization	Control (n = 55)	Intervention (n = 55)
n (%)		
Male	28 (50.91)	25 (45.45)
Female	27 (49.09)	30 (54.55)
Smoking status	15 (27.3)	12 (21.8)
Mean (SD)		
Age (yr)	63.9 (2.2)	63.5 (2.2)
BMI	22.5 (2.2)	23.2 (2.4)
SBP (mm Hg)	140.2 (10.7)	142.9 (8.5)
DBP (mm Hg)	78.6 (6.8)	76.5 (7.8)
FIB (g/L)	6.82 (0.26)	6.9 (0.27)
DD (μg/L)	597.7 (12.69)	596.37 (13.06)
PT (s)	10.69 (0.45)	11.03 (0.42)
APTT (s)	27.53 (0.66)	28.62 (0.72)
TT (s)	11.84 (0.59)	12.05 (0.43)
HCT (%)	0.55 (0.02)	0.54 (0.02)
PLT (×10^9^/L)	301.8 (16.04)	298.37 (14.69)
Hcy (μmol/L)	24.9 (4.3)	26.2 (3.3)
NO (μmol/L)	26.3 (4.5)	25.9 (6.0)
ET-1 (ng/L)	72.3 (5.0)	73.1 (7.6)

### 3.2 Outcomes of first treatment

All data were compared after 12 weeks of treatment. The prothrombotic state showed no significant difference after the first phase of treatment as compared to pretreatment (*P* > .05), and there was no obvious difference between the 2 groups at the 12-week time point (*P* > .05) (Table 2). However, Hcy and ET-1 showed a significant decrease, and NO showed a significant increase in the treatment group after 12 weeks’ treatment with FA (all *P* < .05).

**Table 2:** Statistical comparison of laboratory data of the first treatment between intervention and control groups.

		Control (n = 55)	Intervention (n = 55)	*P* value
SBP (mm Hg)	Pretreatment	140.2 ± 10.7	142.9 ± 8.5	>.05
	Post-treatment	141.5 ± 8.5	141.8 ± 5.0	>.05
DBP (mm Hg)	Pretreatment	78.6 ± 6.8	74.3 ± 5.8	>.05
	Post-treatment	74.3 ± 5.8	75.8 ± 7.5	>.05
FIB (g/L)	Pretreatment	6.82 ± 0.26	6.9 ± 0.27	>.05
	Post-treatment	6.52 ± 0.18	6.79 ± 0.62	>.05
DD (μg/L)	Pretreatment	597.7 ± 12.69	596.37 ± 13.06	>.05
	Post-treatment	603.1 ± 7.99	600.17 ± 9.60	>.05
PT (s)	Pretreatment	10.69 ± 0.45	11.03 ± 0.42	>.05
	Post-treatment	10.98 ± 0.51	12.03 ± 0.33	>.05
APTT (s)	Pretreatment	27.53 ± 0.66)	28.62 ± 0.72	>.05
	Post-treatment	29.63 ± 4.62	30.60 ± 1.56	>.05
TT (s)	Pretreatment	11.84 ± 0.59	12.05 ± 0.43	>.05
	Post-treatment	12.01 ± 2.12	12.04 ± 0.94	>.05
HCT (%)	Pretreatment	0.55 ± (0.02	0.54 ± 0.02	>.05
	Post-treatment	0.51 ± 0.03	0.49 ± 0.54	>.05
PLT (×10^9^/L)	Pretreatment	301.8 ± 16.04	298.37 ± 14.69	>.05
	Post-treatment	289.8 ± 9.54	288.91 ± 11.41	>.05
Hcy (μmol/L)	Pretreatment	24.9 ± 4.3	26.2 ± 3.3	>.05
	Post-treatment	25.7 ± 3.5	22.1 ± 3.4^∗^	<.05
NO (μmol/L)	Pretreatment	26.3 ± 4.5	25.9 ± 6.0	>.05
	Post-treatment	24.5 ± 5.4	29.1 ± 4.2^∗^	<.01
ET-1 (ng/L)	Pretreatment	72.3 ± 5.0	73.1 ± 7.6	>.05
	Post-treatment	72.4 ± 6.7	69.5 ± 8.6^∗^	<.05

### 3.3 Secondary outcomes

After the first treatment, the dose of FA was adjusted by *MTHFR* C677T genotype. *MTHFR* genotyping showed that 63/110 had the TT genotype (32/55 [58.18%] in the treatment group and 31/55 [56.36%] in the control group). Moreover, 31/110 had the CT genotype (14/55 [25.45%] in the treatment group; 17/55 [30.91%] in the control group). Additionally, 16/110 had the CC genotype (9/55 [16.36%] in the treatment group; 7/55 [12.7%] in the control group). After the first phase, patients were treated with different doses of FA according to the genotype and the manufacturer's instructions: TT genotype, 5 mg; CT genotype, 2.5 mg; and CC genotype, 0.8 mg per day, for another 12 weeks.

BP and Hcy, NO, and ET-1 levels showed statistically significant differences between groups (reflecting vascular endothelial function). There was a significant effect of the *MTHFR* C677T polymorphism (Tables 3 and 4). The levels of Hcy and ET1/ NO were significantly decreased in the treatment group after the second phase, and were lower than those after the first treatment phase and lower than in the control group. These differences were statistically significant (*P* < .01). BP, D-dimer level, and fibrinogen scores were statistically significantly lower after the second phase of treatment (*P* < .01).

**Table 3:** Participants’ *MTHFR* C677 genotype.

Genotype	Control (n = 55)	Intervention (n = 55)
CC n (%)	7 (12.7%)	9 (16.36%)
CT n (%)	17 (30.91%)	14 (25.45%)
TT n (%)	31 (56.36%)	32 (58.18%)

**Table 4:** Statistical comparison of the second treatment, between intervention and control groups.

		Control (n = 55)	Intervention (n = 55)	*P* value
SBP (mm Hg)	First treatment	141.5 ± 8.5	141.8 ± 5.0	>.05
	Second treatment	140 ± 9.8	137.4 ± 3.9^∗^	<.05
DBP (mm Hg)	First treatment	74.3 ± 5.8	75.8 ± 7.5	>.05
	Second treatment	72.5 ± 7.2	70.5 ± 5.5^∗^	<.05
FIB (g/L)	First treatment	6.52 ± 0.18	6.79 ± 0.62	>.05
	Second treatment	4.90 ± 0.28^∗^	2.98 ± 0.22^∗^	<.01
DD (μg/L)	First treatment	603.1 ± 7.99	600.17 ± 9.60	>.05
	Second treatment	597.70 ± 12.69	200.03 ± 14.85^∗^	.000
PT (s)	First treatment	10.98 ± 0.51	12.03 ± 0.33	>.05
	Second treatment	10.29 ± 0.45	14.06 ± 0.38^∗^	<.01
APTT (s)	First treatment	29.63 ± 4.62	30.60 ± 1.56	>.05
	Second treatment	35.65 ± 0.72^∗^	39.60 ± 3.11^∗^	<.01
TT (s)	First treatment	12.01 ± 2.12	12.04 ± 0.94	>.05
	Second treatment	15.12 ± 0.43^∗^	18.09 ± 0.37^∗^	<.01
HCT (%)	First treatment	0.51 ± 0.03	0.49 ± 0.54	>.05
	Second treatment	0.47 ± 0.31	0.39 ± 0.03^∗^	<.01
PLT (×10^9^/L)	First treatment	289.8 ± 9.54	288.91 ± 11.41	>.05
	Second treatment	291.4 ± 6.71	213.37 ± 8.95^∗^	<<.01
Hcy (μmol/L)	First treatment	25.7 ± 3.5	22.1 ± 3.4	<.05
	Second treatment	24.2 ± 3.2	15.8 ± 2.8^∗^	<.01
NO (μmol/L)	First treatment	24.5 ± 5.4	29.1 ± 4.2	<.01
	Second treatment	22.0 ± 3.2	44.6 ± 11.5^∗^	.000
ET-1 (ng/L)	First treatment	72.4 ± 6.7	69.5 ± 8.6	<.05
	Second treatment	74.6 ± 5.5	32.1 ± 48.2^∗^	.000

### 3.4 Safety

No side effects were observed in any patient, and there was no significant difference in the incidence of adverse drug reactions between the 2 groups (*P* > .05).

## 4 Discussion

This was a double-blind, randomized controlled trial of FA based on *MTHFR* C677T genotyping in patients with H-type hypertension. We demonstrated that FA combined with levamlodipine besylate, as compared with levamlodipine besylate alone, could significantly reduce the prothrombotic state, and the levels of Hcy and ET-1 in people with H-type hypertension. In treatment phase I, the participants who received FA (0.8 mg) and levamlodipine besylate maintained a significant decrease in Hcy levels, but this difference was not observed in the control group. Hcy levels have long been regarded as a key factor in vascular endothelial function and BP. In this study, secondary treatment outcomes demonstrated that an individualized treatment of FA according to *MTHFR* C677T genotyping was clearly linked to the prothrombotic state.

Previous studies indicated that people with a TT genotype have high plasma Hcy levels.^[14]^ Hcy, a sulfur-containing nonessential amino acid, is commonly used as a biochemical marker of cardiovascular and cerebrovascular diseases and has some value in the diagnosis of hypertension. Hcy plays a crucial role in biological redox homeostasis. High Hcy levels are associated with the pathophysiology of atherosclerosis, which is known to be associated with a prothrombotic state in individuals with hypertension.^[15,16]^ In addition, high Hcy levels can also enhance the sensitivity to vascular endothelial cell damage and amplify damage in cells by mobilizing intracellular calcium release.^[17,18]^ As FA supplementation can prevent Hcy and ET-1/NO increase,^[19]^ FA supplementation seems to be a reasonable treatment for individuals with H-type hypertension, particularly given that it can be achieved by using a relatively low-priced treatment that is easy to obtain in the community and hospital.

The TT genotype in the treatment group comprised more than two-thirds of patients. They received 96% of the individual dose of FA based on testing *MTHFR* C677T genotyping. FA supplementation was effective in decreasing Hcy and ET-1/NO levels, which is related to the prothrombotic state. This supports the argument that H-type hypertension is controllable, and that the benefits of increasing regular FA intake are sustained during treatment with levamlodipine besylate.

People with a prothrombotic state due to Hcy load have fluctuations in BP.^[8]^ H-type hypertension influences BP and cardiovascular health outcomes.^[20,21]^ Other studies have highlighted that *MTHFR* C677T genotyping is important.^[22,23]^ In a recent study of hypertension, hypertension in a small population of people was found to be related to a prothrombotic state,^[24]^ which suggests that the prothrombotic state is a negative influencing factor in hypertension.^[25]^ In this study, 36/110 participants had high prothrombotic state scores. A previous clinical trial showed that all patients with a prothrombotic state who received individualized FA treatment, as compared with controls, had good clinical results.

No previous double-blind trial had provided FA supplements based on levamlodipine besylate treatment in patients with H-type hypertension with a prothrombotic state. Levamlodipine besylate is a CCB used in hypertension. It affects vascular endothelial cells, increasing their NO content and decreasing their ET-1 content. However, these effects are abrogated in HHcy. This trial was designed to test the effectiveness of FA. Therefore, we recruited patients at the time point that was appropriate for each individual within the cure process. This meant that participants were given FA at different doses according to genotype. Several studies have shown that the effects of FA supplementation is dependent on the FA dose as well as the initial serum Hcy and FA concentrations. People with higher levels of folate in their blood fare better in atherosclerosis tests.^[26]^

The study had some limitations. The study had a short follow-up period. The research relied on patients’ reports of taking the medication, which may influence clinical results. Dietary information was provided by the participants. Additionally, there were some differences in baseline factors, including differences in age, sex, and living habits. Dietary assessments were not performed in the trial. However, food FA and supplements have different effects in people. Moreover, there is a need for a medication event monitoring system, but such systems are costly and have disadvantages. Future studies should standardize the dose of FA provided according to each genotype, but should also account for other confounders, such as dietary intake, because these points also influence clinical treatment results. Strengthening the efficiency of hypertension control fundamentally reduces the prothrombotic state of patients, and should be considered in future research.

We aimed to collect data on the prothrombotic state and H-type hypertension. However, some patients did not meet with the researcher to assess clinical effects related to the prothrombotic state. Similarly, some patients were unwilling to continue taking medication throughout their lives. Consequently, some data during the therapeutic process were missing. In future research, we will consider dropout rates in the region of 20% to 25%. Clinical outcomes, which are part of routine cure processes, may be used to determine the prothrombotic state in individuals with H-type hypertension. This may include gene testing to individualize treatment.

## 5 Conclusions

Based on this study, FA combined with levamlodipine besylate can decrease Hcy and ET-1 levels, and can increase NO levels, and can thereby have positive effects on a prothrombotic state and atherosclerosis. This in turn can improve clinical outcomes in people with H-type hypertension. The findings of this clinical randomized, controlled trial are encouraging in that we showed a decrease in the prothrombotic state with H-type hypertension by individualizing FA supplementation. This supports the use of FA in people with H-type hypertension and concurs with other studies promoting optimization of treatment of cerebrovascular and cardiac disease and rehabilitation.^[27]^ FA treatment individualization in people with hypertension requires genotyping by means of a gene test kit and then implementing individualized FA doses. Individualized FA treatment is needed for patients receiving neoadjuvant treatments because their treatment duration is longer than that for patients taking antihypertensive drugs.

## Acknowledgments

We would like to thank Editage (www.editage.com) for English language editing.

## Author contributions

**Conceptualization:** Shuangming Ran, Ruohan Yang.

**Data curation:** Jianping Tang, Ailin Hou, Xiaoling Yan, Baozhong Yu.

**Formal analysis:** Xiaoling Yan, Min Luo, Ying Tang.

**Funding acquisition:** Huaiqi Wang.

**Investigation:** Tianxun Wang, Jianping Tang, Min Luo, Ying Tang, Ruohan Yang, Dongsheng Song.

**Methodology:** Huaiqi Wang.

**Project administration:** Song Zhang, Hanjun He.

**Software:** Ailin Hou, Baozhong Yu.

**Supervision:** Tianxun Wang, Shuangming Ran.

**Validation:** Xiaoling Yan.

**Visualization:** Ruohan Yang.
